# Niobium-Doped Nanofiber Reinforcement of Low-Viscosity Bulk-Fill Resin Composites: Physicomechanical Properties and Mineral Deposition Potential

**DOI:** 10.3390/polym18172170

**Published:** 2026-09-05

**Authors:** Mariana Souza Rodrigues, Tatiana Rita de Lima Nascimento, Rafael Francisco Lia Mondelli, Abdulaziz Alhotan, Saleh Alhijji, Nair Cristina Brondino, Marilia Mattar de Amoêdo Campos Velo

**Affiliations:** 1 Department of Restorative Dentistry, Araraquara School of Dentistry, Sao Paulo State University (FOAr-UNESP), Humaitá, 1680, Araraquara 14801-903, SP, Brazil; 2 Institute for Emerging Electronic Technologies (IET), Leibniz Institute for Solid State and Materials Research, 01069 Dresden, Germany; 3 Department of Operative Dentistry, Endodontics and Dental Materials, Bauru School of Dentistry, University of São Paulo (FOB/USP), Alameda Dr. Octávio Pinheiro Brisolla, 9-75, Vila Universitária, Bauru 17012-901, SP, Brazil; 4 Department of Dental Health, College of Applied Medical Sciences, King Saud University, Riyadh 12372, Saudi Arabia; 5 Department of Mathematics, Faculty of Sciences of Bauru, São Paulo State University (UNESP), Avenida Eng. Luiz Edmundo Carrijo Coube, 14-01, Bauru 17033-360, SP, Brazil

**Keywords:** polymer nanocomposite, niobium nanofibers, bulk-fill resin, bioactive materials, dental polymers

## Abstract

**Background:** Bulk-fill resin composites have limitations regarding depth of cure and often lack bioactive functionality, which may compromise their long-term clinical performance. **Purpose**: This in vitro study evaluated the effects of incorporating niobium-doped nanofibers (Nb-NFs) on the physicomechanical properties and bioactive potential of an experimental low-viscosity bulk-fill resin composite. **Methods:** Three groups were investigated: BF (experimental resin; negative control), BFflow-fiber (experimental resin containing 1 wt% Nb-NFs), and S-PRG (commercial bioactive resin; positive control). Surface hardness (SH) was measured on the top and bottom surfaces using Knoop microhardness testing (*n* = 6), and depth of cure (DoC) was calculated as the bottom-to-top hardness ratio, with a value of ≥80% considered acceptable. Surface roughness (SR; *n* = 6) was assessed using profilometry. Mineral deposition was analysed using X-ray diffraction (XRD) and Fourier-transform infrared spectroscopy (FTIR) after 21 days of immersion in simulated body fluid. The data were analysed using analysis of variance (ANOVA) and Tukey’s test (*p* < 0.05). **Results:** The incorporation of Nb-NFs significantly increased SH on both surfaces (*p* < 0.05) but reduced DoC (*p* < 0.05). FTIR revealed phosphate bands characteristic of calcium phosphate formation, while XRD indicated initial apatite nucleation. Surface roughness remained comparable to that of the control groups. **Conclusions:** Nb-NFs represent a promising reinforcement strategy for bioactive bulk-fill composites; however, further optimisation is required to improve the depth of cure before clinical application.

## 1. Introduction

Although conventional resin composites have demonstrated satisfactory clinical performance [1], their placement using the incremental technique remains technique-sensitive and time-consuming, as multiple layering and photoactivation steps are required to ensure adequate polymerisation [2]. To overcome these limitations, bulk-fill resin composites were developed to permit placement in increments of 4–5 mm while maintaining acceptable polymerisation throughout the restoration [3].

These materials simplify clinical procedures through compositional modifications, including increased translucency, optimised photoinitiator systems, and modified filler characteristics, which enhance light transmission and promote a greater depth of cure (DoC) [4,5,6]. Consequently, this approach reduces chair time, minimises operator-dependent variability, and improves clinical efficiency [5,6]. Bulk-fill resin composites are commercially available in both low- and high-viscosity formulations, with low-viscosity variants exhibiting enhanced flowability that facilitates their adaptation to deep cavities and complex geometries [4].

Despite these advantages, concerns remain regarding DoC and its effects on the physicomechanical performance of bulk-fill restorations, particularly in deeper regions. An inadequate DoC may compromise polymerisation at the bottom of the restoration, resulting in inferior mechanical properties, postoperative sensitivity, and premature failure [7,8]. This limitation is particularly relevant to low-viscosity bulk-fill (BFflow) materials, which have been associated with lower surface hardness (SH) than higher-viscosity or conventional resin composites [9].

Beyond these material-related limitations, the long-term success of resin composite restorations is also influenced by clinical and biological factors. Dental caries is a multifactorial process driven by interactions among biofilm activity, dietary habits, and host-related factors, rather than by the restorative material alone [10,11,12,13]. These mechanisms are also responsible for the development of carious lesions adjacent to restorations, commonly referred to as secondary caries, which constitute a major cause of restoration failure [11,14]. Although restorative materials do not directly determine the development of carious lesions adjacent to restorations, their long-term clinical performance may influence the preservation of the tooth–restoration interface. Properties such as marginal adaptation, interfacial sealing, resistance to degradation, and physicomechanical stability may affect biofilm retention and the progression of demineralisation adjacent to restorations [15]. Therefore, irrespective of the resin composite category, materials capable of maintaining interfacial integrity and exhibiting bioactive potential may contribute to improving restoration longevity and reducing degradation over time.

In view of these considerations, the development of restorative materials that combine adequate physicomechanical performance with bioactive functionality has become a central focus of dental materials research [16]. Bioactive systems can interact with the surrounding environment by releasing ions, modulating local pH, and promoting mineral deposition [17,18]. However, achieving these effects without compromising mechanical performance remains a considerable challenge. Various strategies have therefore been proposed, including modifications to filler composition and the incorporation of ion-releasing particles into resin composites. Among these approaches, surface pre-reacted glass-ionomer (S-PRG) technology has been widely investigated because of its capacity to release multiple ions, including fluoride, strontium, and silicate, thereby contributing to antibacterial activity, acid neutralisation, and remineralisation [19,20,21]. Nevertheless, S-PRG-based materials may still have limitations in terms of mechanical performance and long-term stability [22,23,24,25,26].

More recently, reinforcement strategies based on nanostructured systems, particularly nanofibers, have attracted attention because of their potential to improve stress distribution, enhance interfacial bonding, and strengthen resin-based materials [27,28,29,30]. Previous studies have demonstrated that hybrid nanofibers doped with niobium (Nb) nanoparticles can significantly improve physicomechanical properties while promoting a more homogeneous distribution of fillers within the polymer matrix [25,31]. In addition to their reinforcing effects, niobium pentoxide (Nb_2_O_5_) exhibits favourable physicochemical properties, including high chemical stability and biocompatibility [25]. Furthermore, niobium oxide compounds have been associated with hydroxyapatite nucleation and mineral deposition in biological environments, indicating their bioactive potential. Studies have shown that niobium-containing materials can promote apatite formation on their surfaces when exposed to simulated body fluids, suggesting their potential to facilitate interfacial mineralisation in restorative systems [23,32,33,34,35].

Despite the promising findings reported for niobium-containing systems in conventional resin-based materials [18,31], their behaviour in BFflow composites remains insufficiently explored. Because of their lower filler content, these materials require a careful balance among handling characteristics, physicomechanical performance, and long-term stability [7,8,9]. From a clinical perspective, this balance is particularly important because BFflow composites are frequently used in deep cavities and minimally invasive restorations, in which longevity depends on the preservation of surface integrity and the tooth–restoration interface. Although bioactive fillers may promote mineral deposition and contribute to interfacial stability, it remains unclear whether the incorporation of Nb-nanofibers can provide these benefits without adversely affecting clinically relevant properties such as DoC, SH, and surface roughness (SR).

Considering the need to improve both the physicomechanical performance and bioactive behaviour of low-viscosity bulk-fill materials, the incorporation of Nb-nanofibers represents a promising strategy. Therefore, the aim of this in vitro study was to incorporate Nb-nanofibers into an experimental BFflow composite and evaluate its physicomechanical properties and mineral deposition potential. The hypothesis was that the incorporation of Nb-nanofibers would enhance physicomechanical performance and mineral deposition without compromising DoC or surface properties.

## 2. Materials and Methods

### 2.1. Materials

Bulk-fill resin composites modified with nanofibers were synthesised and characterised. The type of resin composite was considered the study factor and comprised three levels: BF (experimental BFflow resin composite), BFflow-fiber (experimental BFflow resin composite containing 1 wt% niobium-doped nanofibers), and S-PRG (commercial BFflow resin composite containing bioactive S-PRG fillers; Beautiful Bulk Restorative, Shofu) (Table 1). The primary outcomes were surface hardness (SH) on the top and bottom surfaces, depth of cure (DoC), calculated from the bottom-to-top hardness ratio, and surface roughness (SR). Mineral deposition was also analysed using X-ray diffraction (XRD) and Fourier-transform infrared spectroscopy (FTIR). The number of specimens per group was *n* = 6 for the physicomechanical tests and *n* = 3 for the qualitative mineral deposition analysis.

### 2.2. Synthesis of Niobium-Doped Nanofibers and Experimental Resin Composite Preparation

The niobium-doped nanofibers used in this study were synthesised according to the protocol described by Velo et al. [31]. Niobium nanofibers were incorporated exclusively into the experimental resin of the BFflow-fiber group at a concentration corresponding to 1% of the total mass of the experimental resin (1 wt%), while BF and S-PRG were maintained as comparative controls. The experimental resin composite was weighed using a precision analytical balance with a readability of 0.000 g (Adventurer Precision AX, OHAUS Corporation, Parsippany, NJ, USA), and the amount of Nb-nanofibers required to achieve the designated weight percentage was calculated. The mixture was subjected to magnetic stirring for 30 min [23] to further promote dispersion and stability of the nanofibers within the resin matrix. Specimens were fabricated using a non-split cylindrical stainless-steel mold measuring 4 mm in diameter and 4 mm in height. The specimens used for surface roughness analysis measured 15 × 1 mm, according to the respective ISO 4049 [36] standards. Photoactivation was performed for 40 s using a VALO Grand light-curing unit (Ultradent, South Jordan, UT, USA), operating at a wavelength range of 385–515 nm and an irradiance of 1200 mW/cm^2^. The light-curing tip was positioned perpendicular to and in direct contact with the specimen surface (0 mm distance). Irradiance was verified using a radiometer before specimen fabrication. After specimen preparation, all °C testing was in accordance with standard protocols for resin-based materials (ISO 4049) [36].

### 2.3. Samples Size Calculation

For the sample size calculation, top-surface SH was defined as the primary outcome. Reference values were derived from pilot data, in which the S-PRG group (positive control) had a mean top-surface SH of 41.09 and a standard deviation of 1.57. Based on these data, the BFflow-fiber group was assumed to exhibit a mean hardness value 10% higher than that of the S-PRG group, whereas the BF group was assumed to exhibit a value 10% lower. R software 4.5.1 (R Core Team, 2025) was used to calculate the sample size, assuming α = 0.05, a statistical power of 0.80, and equal standard deviations (1.57) across groups. A Monte Carlo simulation with 5000 iterations was performed to assess the robustness of the estimated sample size, indicating a statistical power greater than 0.90 under the specified conditions. The remaining outcomes were also considered primary variables; however, due to the lack of prior data, no separate sample size calculation was performed. For these outcomes, effect sizes and their corresponding 95% BCa confidence intervals, based on 5000 bootstrap replications, will be reported to provide information on the magnitude and uncertainty of the observed effects. The four responses represent distinct, prespecified scientific hypotheses and were therefore analyzed separately. Nevertheless, because all four were designated as primary outcomes, multiplicity across the four primary hypotheses was addressed using the Holm procedure. Within each outcome, pairwise comparisons among the three groups were adjusted separately.

### 2.4. Surface Hardness (SH)

Surface hardness (SH) was measured on the top and bottom surfaces of the specimens (*n* = 6) after storage in an incubator at 37 °C for 24 h. Knoop microhardness testing was performed by a single operator using a MicroMet 6040 microhardness tester (Buehler Ltd., Lake Bluff, IL, USA). A diamond indenter was applied with a load of 50 g for 15 s [37]. Three indentations, spaced 100 µm apart, were made on both the top and bottom surfaces of each specimen. The mean of the three measurements was calculated for each surface.

### 2.5. Estimated Depth of Cure (DoC) Analysis

Depth of cure (DoC) was calculated as the percentage ratio of the bottom-surface Knoop hardness value to the top-surface value. A bottom-to-top microhardness ratio of at least 80% was considered indicative of adequate polymerisation [3].

### 2.6. Surface Roughness (SR) Analysis

Surface roughness (SR) was evaluated for all groups (*n* = 6) using a Surftest SJ-400 digital profilometer (Mitutoyo, Kanagawa, Japan). The following measurement parameters were used: minimum tolerance (Tmin) = 0.01 µm; maximum tolerance (Tmax) = 8.00 µm; tracing length (Lt), corresponding to the distance travelled by the stylus, =1.5 mm; cut-off length (Lc), corresponding to the filtering limit or wavelength, =0.25 mm; and measurement length (Lm), corresponding to the effective length considered in the reading, =1.25 mm [38]. The mean of three readings obtained along the x- and y-axes was calculated and recorded as the final SR value for each specimen.

### 2.7. In Vitro Immersion Test in Simulated Body Fluid (SBF)

The mineral deposition capacity of the materials was evaluated according to the methodology described by Obeid et al. [34]. Disc-shaped specimens (14 mm × 1 mm; *n* = 3) were individually immersed in 5 mL of simulated body fluid (SBF), prepared according to Kokubo’s formulation. The SBF contained sodium chloride (NaCl), potassium chloride (KCl), dipotassium hydrogen phosphate trihydrate (K_2_HPO_4_·3H_2_O), magnesium chloride hexahydrate (MgCl_2_·6H_2_O), calcium chloride (CaCl_2_), sodium sulphate (Na_2_SO_4_), tris(hydroxymethyl)aminomethane (Tris), and 1 M hydrochloric acid (HCl), with the pH adjusted to 7.4 [34]. The specimens were stored individually in sealed containers at 37 °C, and the SBF solution was renewed every 72 h throughout the immersion period [34]. The evaluation time points were defined as T0 (baseline) and T21 (after 21 days of immersion). The specimens were subsequently analysed using Fourier-transform infrared spectroscopy (FTIR) and X-ray diffraction (XRD).

### 2.8. Fourier Transform Infrared Spectroscopy (FTIR) and X-Ray Diffraction (XRD)

To evaluate mineral deposition, Fourier-transform infrared spectroscopy (FTIR) analysis was performed. Specimens from each group were analysed at T0, before immersion in simulated body fluid, and at T21, after 21 days of immersion. Spectra were obtained over the range of 4000–400 cm^−1^. The peaks were assigned to the vibrational modes of the PO_4_^3−^, OH^−^, CO_3_^2−^, Nb–O, and Nb=O functional groups.

X-ray diffraction (XRD) analysis was performed to evaluate changes in the specimens and identify the presence of crystalline structures following immersion. Diffraction patterns were recorded over a 2θ range of 5–120° using Cu Kα radiation, with a step size of 0.02° and a counting time of 40 s per step. The operating conditions were set at 50 kV and 40 mA. The presence of characteristic peaks corresponding to hydroxyapatite and Ca(OH)_2_ was assessed. The diffraction patterns generated through the interaction of X-rays with the crystalline materials enabled the identification of the crystalline phases present in the specimens.

### 2.9. Statistical Analysis

Data for SH on the top and bottom surfaces, DoC, and SR were statistically analysed using R software (R Core Team, 2025). Top hardness was analyzed using one-way ANOVA, whereas base hardness and depth of cure were analyzed using generalized linear models. Surface roughness was analyzed using weighted regression. Tukey’s test was used for all pairwise comparisons. Residual distributional assumptions were assessed using Q–Q plots with simulated envelopes and plots of residuals versus predicted values. Homogeneity of variances was additionally assessed using Levene’s or Brown–Forsythe tests. Pairwise comparisons between groups were carried out using Tukey’s test. All analyses were conducted with a significance level of a = 5%.

## 3. Results

### 3.1. Top Surface Hardness (SH)

**Figure 1:**
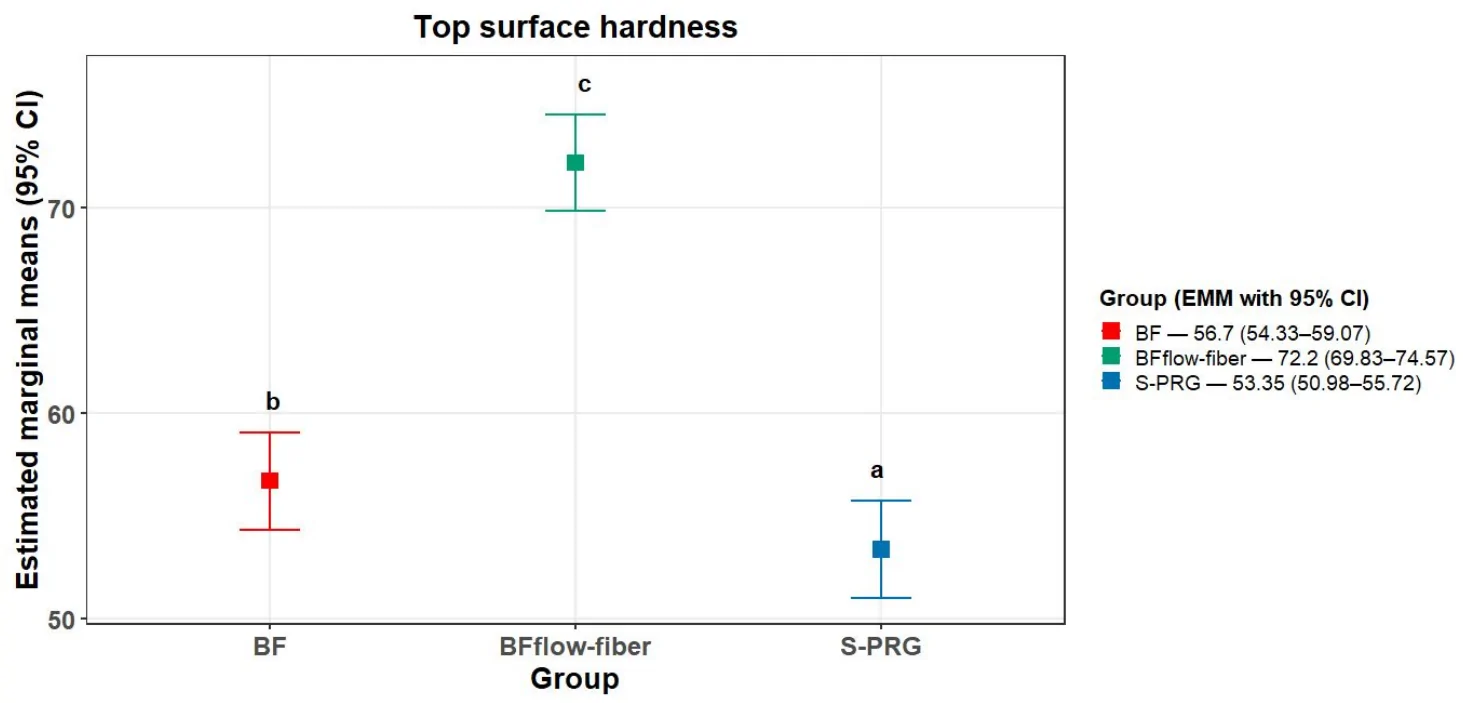
Mean and standard deviation of surface hardness at the top surface of the specimens. Different lowercase letters indicate statistically significant differences between groups (*p* < 0.05).

No substantial deviations from normality or homoscedasticity were observed, and Levene’s test indicated no evidence of heteroscedasticity (*p* = 0.61). ANOVA results indicated a significant difference in mean values among the groups (Holm-adjusted *p* < 0.0001), leading to rejection of the null hypothesis of equal means. The effect size was very large (ω^2^ = 0.94; 95% BCa CI: 0.742–0.964), indicating that approximately 94% of the variability in the outcome was attributable to the group effect. The confidence interval suggests that the true effect size is likely to be substantial, with plausible values ranging from 0.742 to 0.964. Figure 1 presents the top-surface SH values for the evaluated groups. The BFflow-fiber group exhibited significantly higher top-surface microhardness than both BF and S-PRG (*p* < 0.05). BF also showed significantly higher microhardness than S-PRG (*p* < 0.05). Thus, all groups differed significantly from each other.

### 3.2. Bottom Surface Hardness (SH)

**Figure 2:**
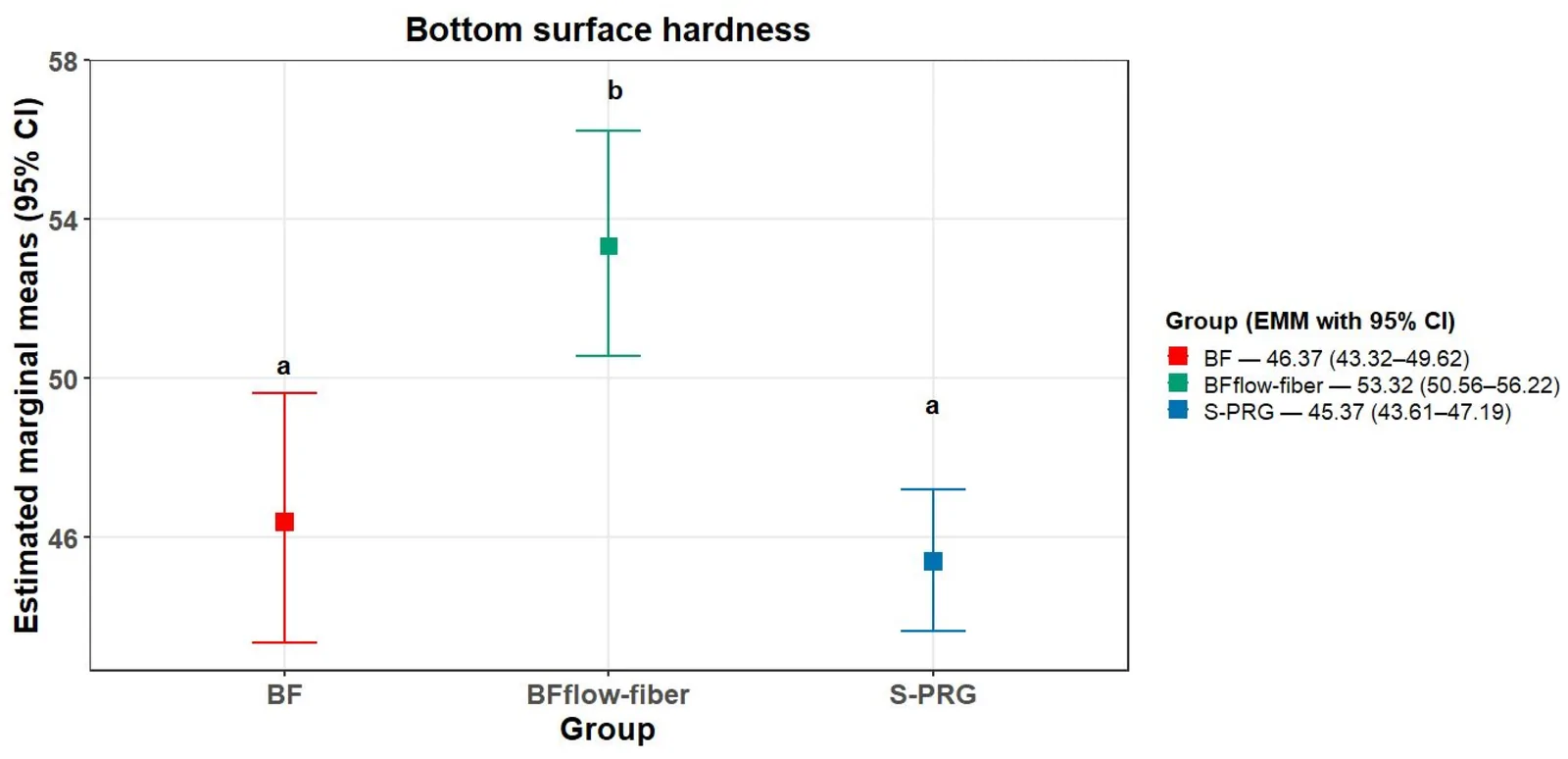
Mean and standard deviation of surface hardness at the bottom surface of the specimens. Different lowercase letters indicate statistically significant differences between groups (*p* < 0.05).

The Q-Q Plot with simulated envelope suggested deviations from normality and the plot of residuals versus predicted values suggested heteroscedasticity, which was supported by the Brown–Forsythe test (*p* = 0.0004). Because the assumptions of normality and homoscedasticity were not adequately met, a generalized linear model with a Gamma distribution and log link function was fitted for bottom SH. The ANDEV results indicated a significant difference in mean values among the groups (Holm-adjusted *p* < 0.0001), leading to rejection of the null hypothesis of equal means. The estimated effect of group on the variability was large (ω^2^ = 0.63), although the BCa 95% CI (0.186–0.809) indicates considerable uncertainty in the estimated magnitude. Figure 2 presents the estimated marginal means of bottom-surface microhardness for the evaluated groups. BFflow-fiber showed significantly higher values than both BF and S-PRG (*p* < 0.05), whereas BF and S-PRG did not differ significantly from each other.

### 3.3. Estimated Depth of Cure (DoC) Results

**Figure 3:**
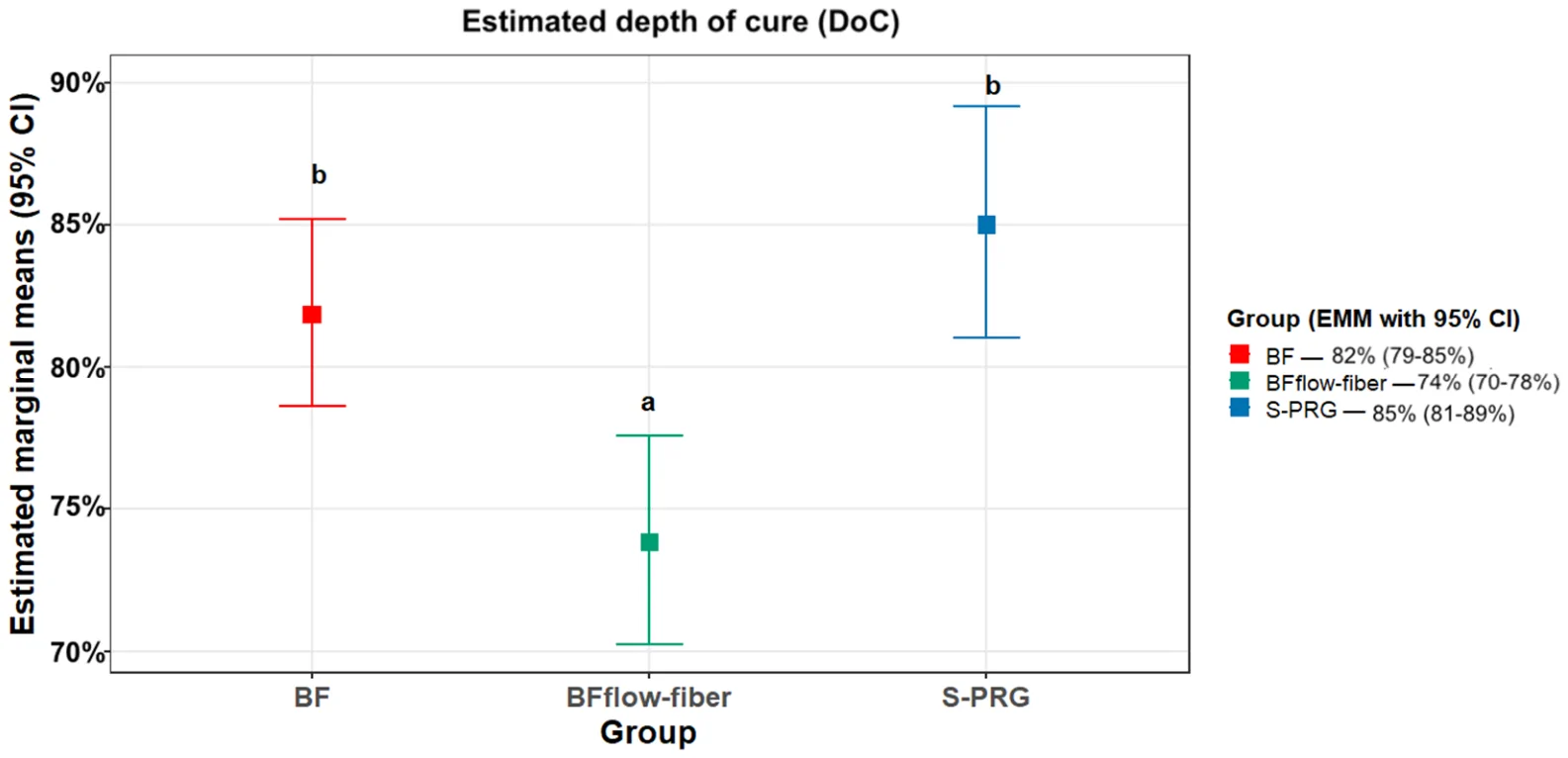
Mean and standard deviation of the depth of cure. Different lowercase letters indicate statistically significant differences between groups (*p* < 0.05).

The Q-Q Plot with simulated envelope suggested deviations from normality and the plot of residuals versus predicted values suggested heteroscedasticity, which was supported by the Brown–Forsythe test (*p* = 0.0003). Because the assumptions of normality and homoscedasticity were not adequately met, a generalized linear model with a Gamma distribution and log link function was fitted for depth of cure. The ANDEV results indicated a significant difference in mean values among the groups (Holm-adjusted *p* < 0.0001), leading to rejection of the null hypothesis of equal means. The estimated effect of group was large (ω^2^ = 0.60), and the BCa 95% CI (0.252–0.764) indicates a consistently substantial effect despite some uncertainty in its magnitude. Figure 3 shows the estimated depth of cure for the evaluated groups. The BFflow-fiber group presented significantly lower estimated depth of cure values than both the BF and S-PRG groups (*p* < 0.05), whereas no significant difference was observed between BF and S-PRG. Notably, the estimated depth of cure of the BFflow-fiber group was below the 80% threshold commonly used as an indicator of adequate polymerization based on the bottom-to-top hardness ratio [3].

### 3.4. Surface Roughness (SR) Results

**Figure 4:**
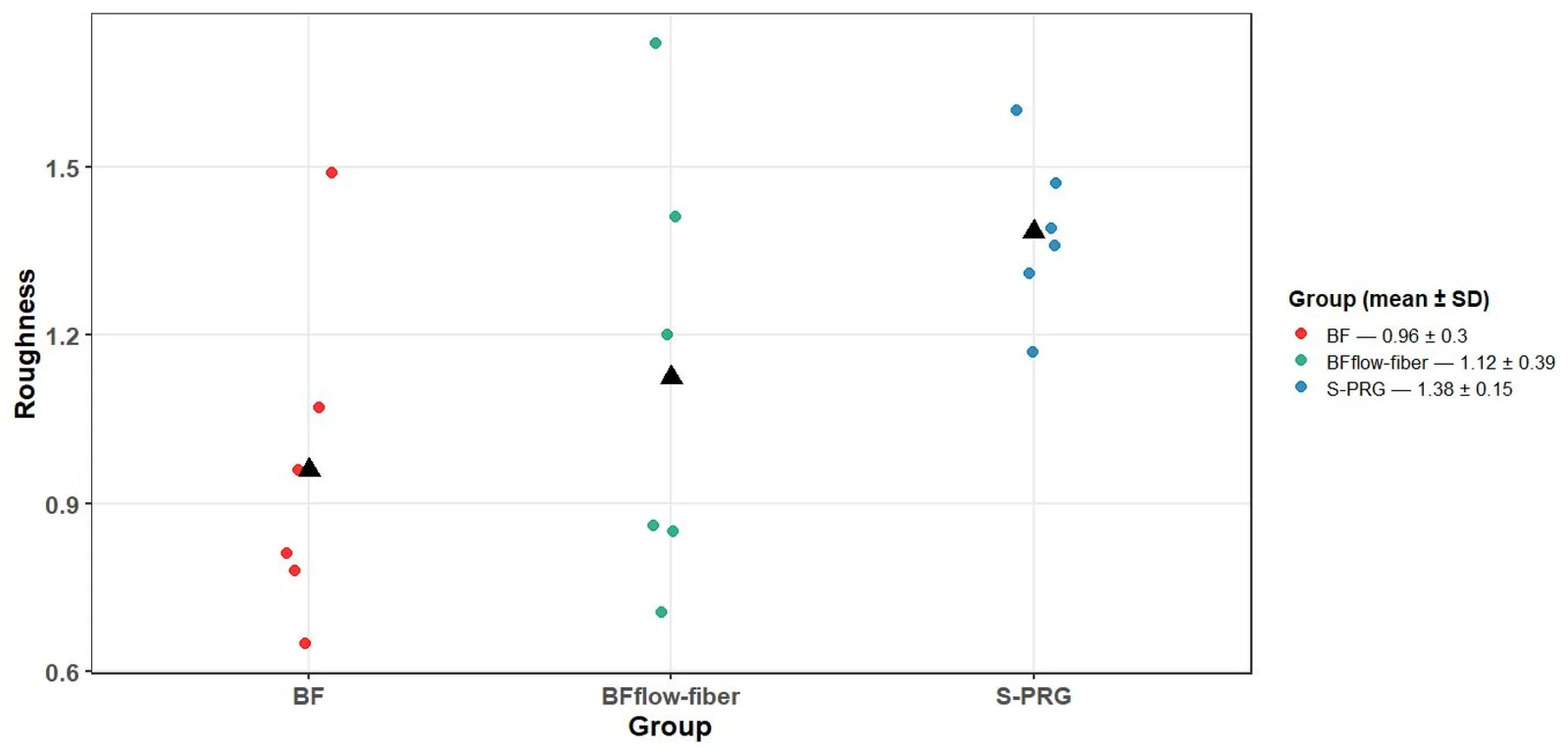
Individual surface roughness values of the groups, including mean and standard deviation.

The residuals were approximately normally distributed and, although Levene’s test did not indicate significant differences in variances among groups (*p* = 0.14), visual inspection of the plot of residuals versus predicted values suggested heteroscedasticity. Therefore, a weighted least-squares regression model was fitted, with weights inversely proportional to the squared fitted absolute residuals. ANOVA indicated a significant difference in means across groups (Holm-adjusted *p* = 0.014), leading to rejection of the null hypothesis of equal means. The point estimated of group effect is moderate (ω^2^ = 0.19); however, the BCa 95% CI (−0.107–0.544) included zero, indicating considerable uncertainty regarding the magnitude of the effect. The individual data plot presented in Figure 4 illustrates the distribution of surface roughness values among the three evaluated groups. The black triangles represent the arithmetic mean for each group. The S-PRG group exhibited the highest mean surface roughness, while the BFflow-fiber group showed the greatest dispersion of values among the specimens. One-way ANOVA revealed a significant difference among the groups (*p* = 0.014). Tukey’s post hoc test indicated a significant difference between the S-PRG and BF groups (*p* = 0.014). No significant differences were observed between the BF and BFflow-fiber groups (*p* = 0.72) or between the BFflow-fiber and S-PRG groups (*p* = 0.36).

### 3.5. FTIR Analysis

**Figure 5:**
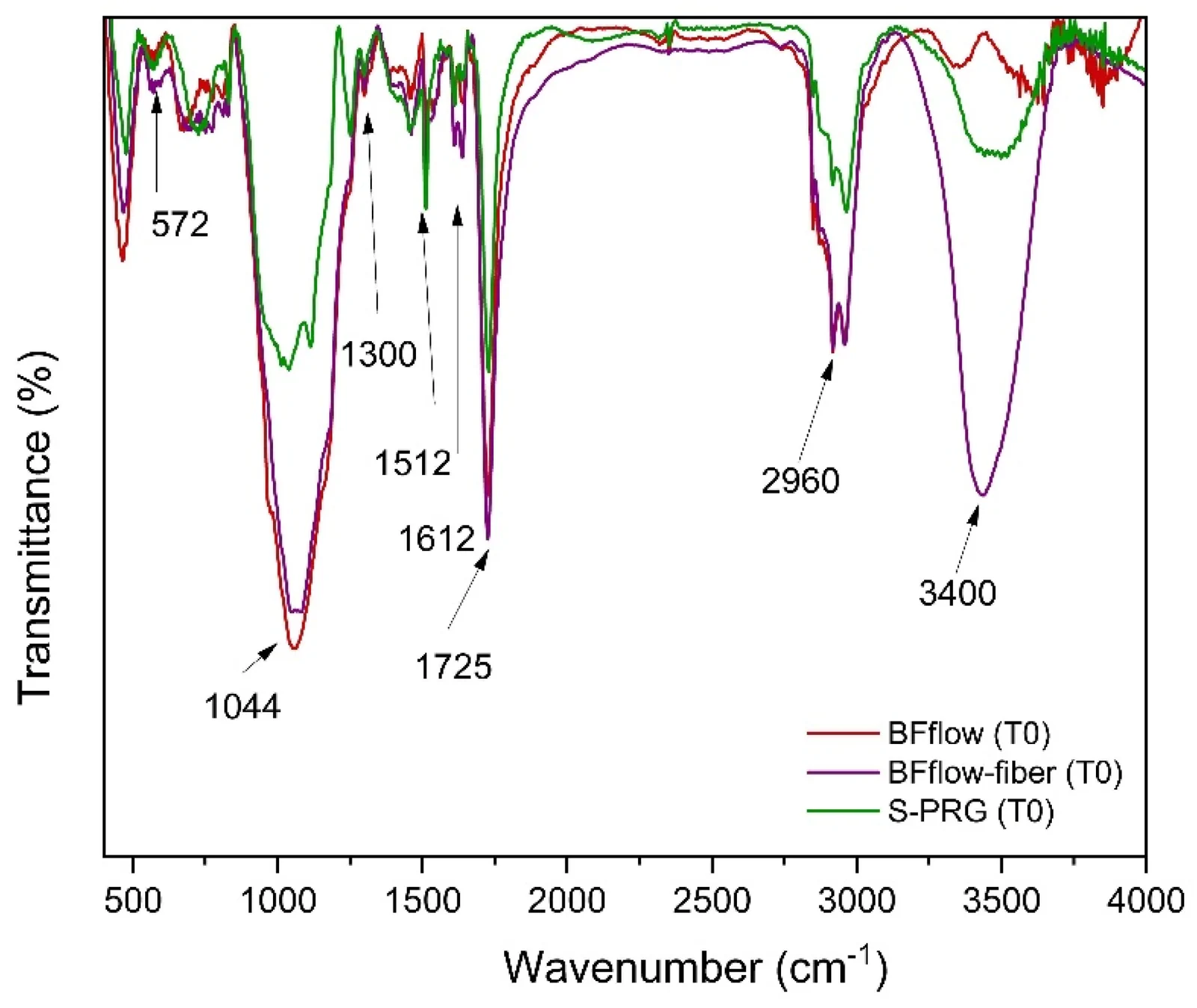
FTIR spectral values at baseline (T0).

**Figure 6:**
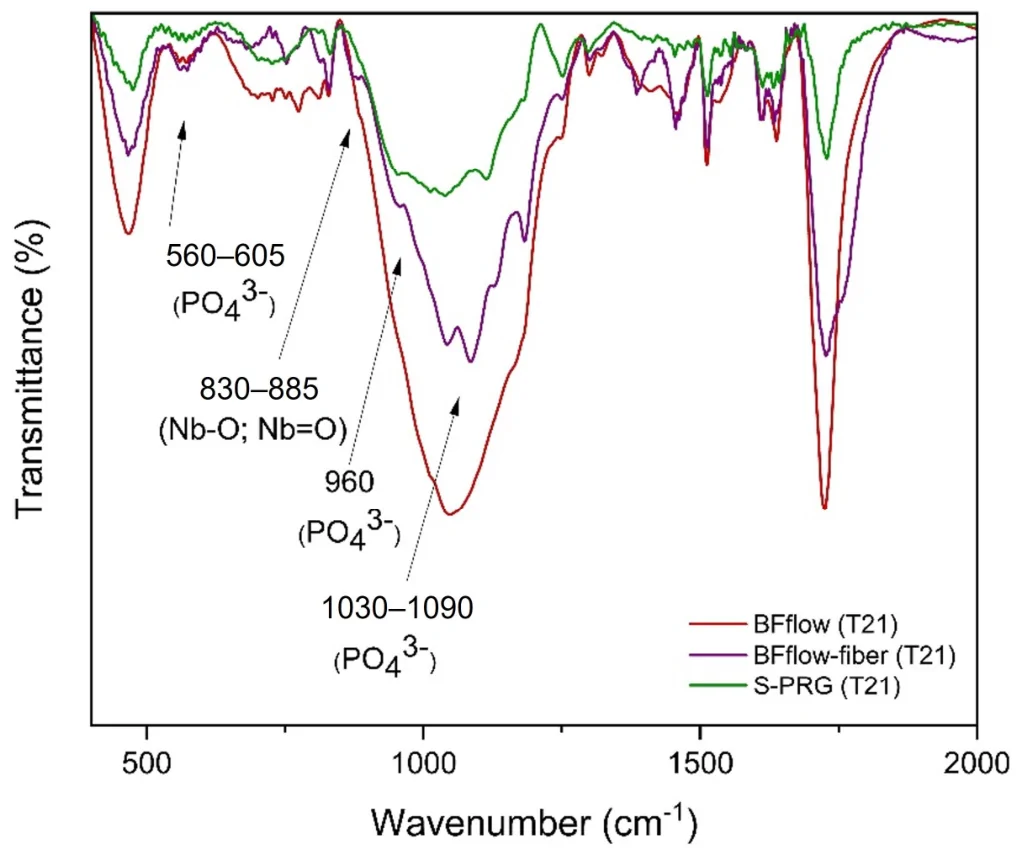
FTIR spectral values after 21 days of immersion in solution body fluid (T21).

Figure 5 and Figure 6 present the FTIR spectra obtained at T0 and T21, respectively. The spectra of the BFflow-fiber specimens revealed characteristic vibrational bands indicative of chemical interactions between phosphate ions (PO_4_^3−^) and the niobium species incorporated into the material. The intensified bands in the regions of 560–605 cm^−1^, ~960 cm^−1^, and 1030–1090 cm^−1^ are consistent with vibrational modes associated with PO_4_^3−^ groups, suggesting the formation of phosphate-containing mineral phases after SBF immersion.

Distinct bands were also identified at 750, 815, 830, and 885 cm^−1^. These bands, which were absent from the control specimens, were attributed to Nb–O vibrations and Nb=O bonds.

### 3.6. X-Ray Diffraction Analysis

**Figure 7:**
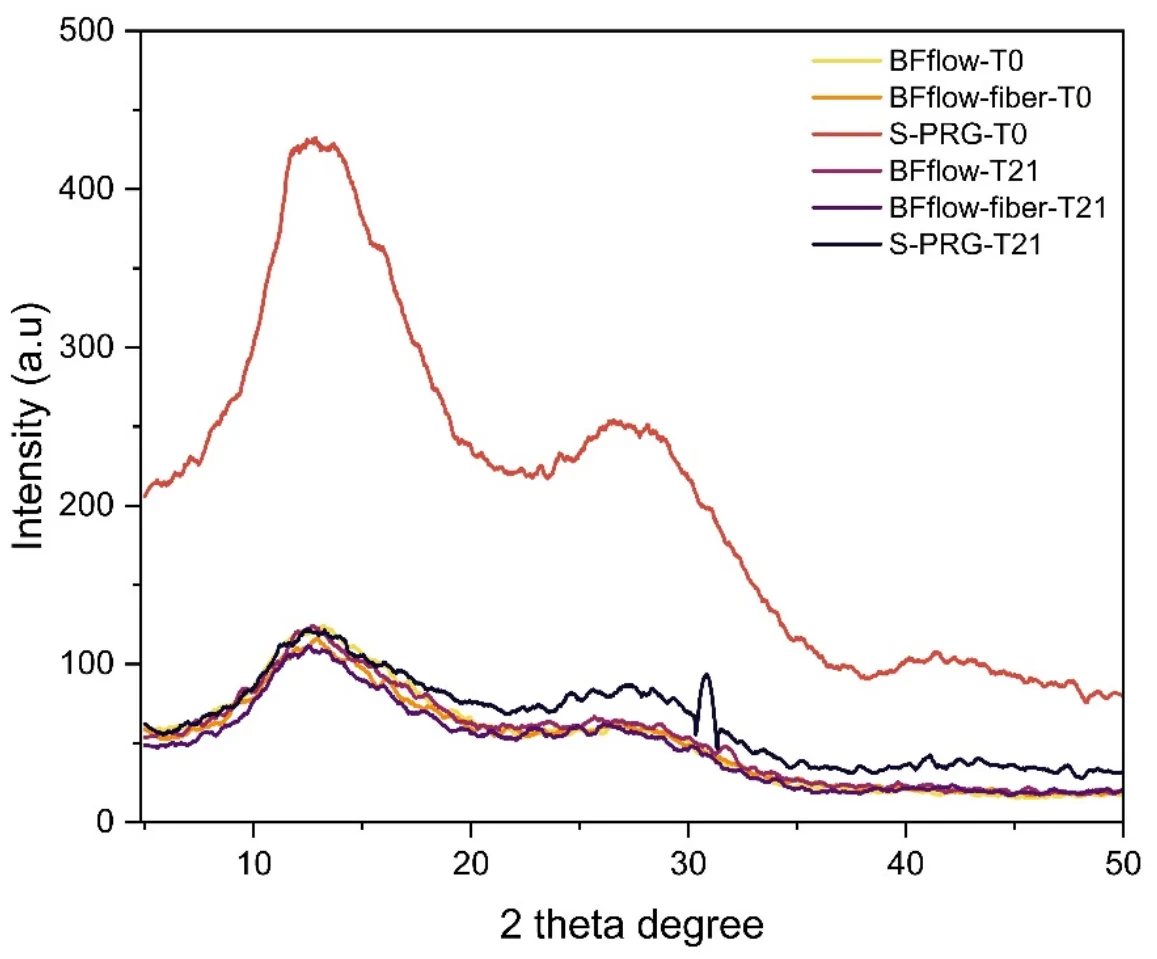
X-ray diffraction (XRD) patterns of the tested materials before (T0) and after 21 days of immersion in simulated body fluid (SBF) (T21).

The X-ray diffraction patterns of the evaluated resin composites are presented in Figure 7. All groups exhibited a broad amorphous halo in the approximately 10–20° 2θ region, characteristic of the resinous polymer matrix. This profile indicates that the incorporation of nanofibers did not result in detectable crystallization of the polymer matrix, which remained predominantly amorphous.

The S-PRG group exhibited markedly greater diffraction intensity than the other groups, which may be attributed to the presence of bioactive fluoroaluminosilicate glass particles. After 21 days of immersion in SBF, a low-intensity diffraction feature was observed around 2θ ≈ 31.7° in the BFflow-fiber group, with an additional feature around 32.0°. These reflections are compatible with apatite-related crystalline phosphate phases. However, given the low intensity of these reflections and the absence of additional phase-specific characterization, the XRD findings should be interpreted as preliminary evidence of the formation of a crystalline phosphate-containing phase rather than definitive identification of hydroxyapatite or carbonated apatite.

## 4. Discussion

The results of this study demonstrated that the incorporation of Nb-nanofibers into a BFflow resin composite influenced its mechanical and physicochemical properties, particularly SH, DoC, and SR, which are important determinants of the clinical longevity of dental restorations [17]. These effects were not uniform throughout the material, indicating depth-dependent behaviour that should be carefully considered in bulk-fill applications. This finding is consistent with previous studies showing that the microhardness and surface characteristics of bulk-fill materials vary according to their composition and light-curing efficiency [39,40]. As the incorporation of Nb-nanofibers improved certain properties but reduced DoC, the study hypothesis was only partially accepted.

For top-surface SH, the BFflow-fiber and BF groups exhibited significantly higher values than the S-PRG group, although they did not differ significantly from each other (Figure 1). At the bottom surface, however, the BFflow-fiber group exhibited significantly higher SH values than both the BF and S-PRG groups (Figure 2). These findings indicate that the incorporation of Nb-nanofibers particularly improved hardness in the deeper region of the material. This behaviour is consistent with previous studies reporting that nanofillers can reinforce the resin matrix and improve stress distribution [41,42]. Similarly, investigations of niobium-containing fillers and nanostructured reinforcing agents have demonstrated improvements in hardness and mechanical performance resulting from enhanced filler–matrix interactions and stress-transfer mechanisms [30,31]. Although the BFflow-fiber group presented an estimated depth of cure of approximately 74% (Figure 3), below the 80% threshold commonly used as an indicator of adequate polymerization, the increased surface hardness observed on both evaluated surfaces suggests that the incorporation of Nb nanofibers may provide a favorable reinforcing effect.

This apparent discrepancy between increased bottom-surface SH and reduced DoC may reflect a complex interaction between light propagation and material composition. The presence of nanofibers may increase internal light scattering, redistribute irradiance within the material, and promote localised polymerisation while reducing effective light transmission through the bulk [43,44]. Consequently, the absolute hardness of deeper regions may increase without a corresponding improvement in the bottom-to-top hardness ratio. Similar behaviour has been reported in highly filled resin systems, in which scattering phenomena alter curing dynamics without improving DoC [5,23]. The present results therefore reinforce the importance of interpreting absolute bottom-surface hardness together with the top-surface value and the resulting hardness ratio, particularly when changes in filler composition affect light transmission [5,23]. Bottom-surface SH should not, therefore, be used as an isolated indicator of adequate polymerisation.

The reduction in depth of cure may be associated with optical differences between the resin matrix and the nanofibers, which could increase light scattering, diffusion, and attenuation. In addition, possible nanofiber agglomeration may act as scattering centers, potentially reducing the irradiance reaching deeper regions and limiting the degree of conversion. These mechanisms were not directly investigated in the present study and should therefore be considered as possible explanations. Nevertheless, these findings are consistent with previous reports showing that filler characteristics can significantly affect light transmission and curing efficiency [5,45].

Regarding SR, the S-PRG group exhibited the highest mean value. It differed significantly from the BF group, whereas the BFflow-fiber group displayed intermediate values and did not differ significantly from either group (Figure 4). These findings indicate that the incorporation of Nb-nanofibers did not significantly increase surface roughness relative to the unmodified experimental bulk-fill material. This behaviour may be attributable to the low concentration of nanofibers used (1 wt%), which may have been insufficient to produce substantial changes in surface topography. Furthermore, because of their nanoscale dimensions, the nanofibers were likely embedded within the resin matrix rather than exposed at the surface, thereby limiting their influence on roughness. The absence of a significant increase in SR may also indicate adequate interaction between the nanofibers and the resin matrix, reducing the occurrence of defects associated with filler debonding or pull-out [31]. In contrast, the higher roughness observed for S-PRG is consistent with previous studies associating ion-releasing bioactive fillers with changes in surface morphology [28,40,42,46,47]. Increased surface roughness may favour plaque accumulation, staining, and wear over time [48]. Therefore, the incorporation of Nb-nanofibers did not appear to compromise this clinically relevant surface property.

The FTIR and XRD findings provide complementary preliminary evidence of mineral phase formation after SBF immersion. The appearance or intensification of phosphate-associated FTIR bands in the BFflow-fiber group, particularly in the regions of 560–605 cm^−1^, ~960 cm^−1^, and 1030–1090 cm^−1^, is consistent with the presence of PO_4_^3−^-containing phases after immersion. The bands observed in the regions associated with Nb–O and Nb=O vibrations are consistent with the presence of niobium-containing species in the composite. However, the present FTIR analysis does not allow definitive identification of specific calcium phosphate phases or establish a direct role of niobium species in mineral nucleation.

The XRD patterns further showed that the BFflow-fiber group remained predominantly amorphous at T0 and T21, with only low-intensity diffraction features emerging around 31.7–32.0° (2θ) after 21 days of SBF immersion [23]. These reflections are compatible with apatite-related crystalline phosphate phases, but their low intensity and the limited number of detectable reflections do not allow unequivocal identification of hydroxyapatite or carbonated apatite. In comparison, the more pronounced crystalline features observed for S-PRG after SBF immersion are consistent with a greater extent of mineral phase formation under the conditions evaluated. Therefore, the present findings suggest that the incorporation of 1 wt.% Nb-containing nanofibers may be associated with early or limited mineral phase formation, although the specific mechanisms underlying this response cannot be established from the present characterization alone.

These findings are consistent with previous reports indicating that niobium-containing materials may be associated with mineral deposition, although the extent and nature of mineral formation may depend on factors such as filler concentration, particle morphology, and exposure time [30,31]. Additional investigations involving ion-release analysis, Ca/P ratio determination, Raman spectroscopy, and models of interfacial remineralization would be necessary to characterize the nature and extent of mineral deposition and to determine its potential biological and clinical relevance. Therefore, the findings of the present study should be regarded as preliminary evidence of mineral phase formation after SBF immersion rather than definitive evidence of remineralization or clinically meaningful bioactivity.

From a clinical perspective, the increase in bottom-surface SH suggests that the incorporation of Nb-nanofibers may improve resistance to indentation in deeper regions of the restoration. Nevertheless, the reduction in DoC raises concerns regarding polymerisation uniformity in bulk-fill applications and may compromise long-term performance [49]. Several studies have demonstrated that DoC is strongly influenced by material translucency, filler characteristics, and curing conditions because these factors directly affect light transmission and polymerisation efficiency in bulk-fill composites [50,51]. In particular, filler size, concentration, refractive index, and light-attenuation mechanisms play critical roles in determining curing effectiveness at greater depths [50]. Optimisation of the nanofiber concentration, dispersion, and optical compatibility with the resin matrix may therefore be required before this approach can be considered suitable for clinical application.

Several limitations should be acknowledged. Although surface roughness is associated with bacterial adhesion, biofilm formation was not directly evaluated; therefore, any biological implications of the SR findings should be interpreted cautiously. Another limitation is that the degree of conversion was also not directly determined using spectroscopic methods such as FTIR or Raman spectroscopy. Instead, polymerisation efficiency was indirectly assessed using top- and bottom-surface hardness measurements and the bottom-to-top hardness ratio, which is an established method for estimating DoC in bulk-fill resin-based materials. Nevertheless, direct assessment of the degree of conversion would provide complementary information regarding the polymerisation behaviour of the experimental resin. Previous research demonstrated that the incorporation of 1 wt% Nb_2_O_5_-filled poly(D,L-lactic acid) nanofibers into a dual-cure resin cement (RelyX U200) did not significantly affect the degree of conversion [31]. However, considering differences in resin composition and polymerization system, these findings cannot be directly extrapolated to the present experimental bulk-fill composite. Therefore, further studies directly evaluating the degree of conversion are warranted to better elucidate the effects of Nb-containing nanofibers on polymerization. In addition, although mechanical performance, water sorption and solubility, aging, and wear resistance are clinically relevant properties of restorative materials, these parameters were not evaluated in the present proof-of-concept study and should be addressed in future investigations.

Mineral deposition was also evaluated using a limited number of specimens (*n* = 3). According to Kokubo and Takadama, immersion tests in SBF are primarily intended as screening methods for evaluating the apatite-forming ability of materials [52]. Accordingly, the present analysis should be interpreted as qualitative, preliminary evidence of phosphate-containing mineral deposition rather than quantitative confirmation of bioactivity. Additional studies employing complementary methods, including scanning electron microscopy coupled with energy-dispersive X-ray spectroscopy, Raman spectroscopy, and ion-release analysis, would enable more comprehensive characterisation of the deposited mineral phase and strengthen the biological interpretation of the findings [53].

Finally, the in vitro design cannot fully reproduce the complexity of the oral environment, particularly with regard to light attenuation in clinically relevant cavity configurations, long-term ageing, masticatory loading, temperature and pH fluctuations, and biological interactions. Accordingly, the clinical potential of the experimental material should be interpreted cautiously at this stage. Future studies should include direct measurements of the degree of conversion, quantitative characterisation of mineral deposition, ion-release testing, biofilm models, interfacial remineralisation analyses, and long-term mechanical and biological evaluations. Different nanofiber concentrations, dispersion methods, resin formulations, restoration depths, and photoactivation protocols should also be investigated to determine whether the reinforcing and mineral-deposition effects of Nb-nanofibers can be achieved without compromising DoC.

## 5. Conclusions

Within the limitations of this in vitro study, the incorporation of 1 wt% Nb-nanofibers into an experimental low-viscosity bulk-fill resin composite increased bottom-surface hardness. It maintained top-surface hardness and surface roughness at levels comparable to those of the unmodified control. However, a reduction in estimated depth of cure was observed, indicating the need for further optimization of resin composition and nanofiber concentration. Preliminary evidence of mineral phase formation was also observed after SBF immersion. Further studies addressing degree of conversion, ion release, long-term stability, and interfacial remineralization are needed before any clinical implications can be established.

## Figures and Tables

**Table 1:** Composition of experimental and commercial bulk-fill flowable resins used in this study.

Material	Group	Composition
Experimental low-viscosity bulk-fill flowable resin	BF	Urethane dimethacrylate, silica, stabilizers, camphorquinone, co-initiator
Experimental low-viscosity bulk-fill flowable resin. + 1%wt Niobium Nanofibers	BF-Nb	Urethane dimethacrylate, silica, stabilizers, camphorquinone, co-initiator, and addition of 1 wt% niobium nanofibers
Commercial resin Beautiful Bulk Flowable (Shofu Inc., Kyoto, Japan)	S-PRG	Bis-GMA, UDMA, Bis-MPEPP, TEGDMA, and S-PRG pre-reacted glass-ionomer filler based on fluoroboroaluminosilicate glass

## Data Availability

The data presented in this study are available on request from the corresponding author.
